# EVALUATION OF PRECLINICAL ALZHEIMER’S DEMENTIA USING PLANTAR PRESSURE REGULATION ABILITY

**DOI:** 10.1093/geroni/igae098.3967

**Published:** 2024-12-31

**Authors:** Jieun Yoon, Kazuki Ota, Tetsuaki Arai, Miyuki Nemoto, Kiyotaka Nemoto, Miho Ota, Mariko Miyashita, Tomohiro Okura

**Affiliations:** Uiversity of Tsukuba, Tsukuba, Ibaraki, Japan; Uiversity of Tsukuba, Tsukuba, Ibaraki, Japan; Institute of Medicine/University of Tsukuba, Tsukuba, Ibaraki, Japan; Tanita Corporation, Itabashi, Tokyo, Japan; Institute of Medicine/University of Tsukuba, Tsukuba, Ibaraki, Japan; Institute of Medicine/University of Tsukuba, Tsukuba, Ibaraki, Japan; Tanita Corporation, Itabashi, Tokyo, Japan; Uiversity of Tsukuba, Tsukuba, Ibaraki, Japan

## Abstract

Strong connectivity between peripheral sensors and the central nervous system assists in cognitive load distribution. Aging and neurological disorders, including various peripheral neuropathies cause decreased plantar skin sensitivity. The early detection and prevention of Alzheimer’s disease, particularly during the preclinical period, is highly important. The preclinical Alzheimer’s cognitive composite 5 (PACC5) test formula is an effective test for assessing the preclinical AD period. However, completing the PACC5 is time consuming and psychologically and physically challenging for 60- to 87-year-old patients. We developed a plantar pressure performance test to evaluate the correlation between PACC5 scores and graded plantar distributions in the preclinical period. Participants (n = 210) aged 60–87 years (mean: 71.0 ± 7.0) provided informed consent. PACC5, gap_total, gap_downslope, gap_upslope, and error (deviation from the target value) values for each relaxing- and exerting-force movement of the legs were evaluated. We used the BM-220 (prototype, Tanita Co. Ltd) for analyzing plantar pressure. The participants were divided into three groups (upper, middle, and lower) based on plantar pressure. Group differences were assessed using Spearman’s correlation and the Kruskal-Wallis tests, with P < 0.05 considered statistically significant. All items showed a weak negative correlation value trend (R = –0.370 to –0.234). PACC5 scores among the upper, middle, and lower groups (differences ordered from lowest to high) showed significant differences, and the PACC5 scores of the lower group were less than those of the upper group. Thus, plantar pressure movement may serve as a preliminary performance index for preclinical period selection.

